# Editorial: Application of Novel Statistical and Machine-Learning Methods to High-Dimensional Clinical Cancer and (Multi-)Omics Data

**DOI:** 10.3389/fgene.2021.739442

**Published:** 2021-08-12

**Authors:** Chao Xu, Shaolong Cao, Md. Ashad Alam

**Affiliations:** ^1^Department of Biostatistics and Epidemiology, University of Oklahoma Health Sciences Center, Oklahoma City, OK, United States; ^2^University of Texas M. D. Anderson Cancer Center, Houston, TX, United States; ^3^Tulane University School of Medicine, New Orleans, LA, United States

**Keywords:** multi-omics analysis, high-dimensional data analysis, cancer genomics, prediction analysis, integrative analysis

The big genomics data from various aspects (e.g., DNA polymorphism, transcriptomics, and proteomics) is now available in cancer research and clinic application. These (multi-)omics data come with a new feature of high dimension: much more features/predictors relative to the available sample size. Meanwhile, researchers are looking beyond individual omics study and exploring integrative analysis of (multi-)omics data. Accordingly, there are novel statistical and machine learning methods designed for the high-dimensional and/or integrative (multi-)omics data analysis. The present Research Topic collects the methodology development and application of statistical and machine-learning methods for high-dimensional clinical (multi-)omics, and integration analysis, mostly, in cancer research.

For multi-omics integration analysis, classical statistical and machine leaning approaches are widely used. Wang et al. did Cox regression analysis on combined immunohistochemical (IHC) markers and synthetic lethal gene pairs. New prognostic markers for Asian oral cancer were reported. Xu et al. used unsupervised cluster-of-clusters analysis to integrate subgroup classification from different omics and identify potential driver genes in cervical cancer. They found four statistically significant expression subtypes by clustering of tumor copy number variation (CNV) and methylation profiles.

New approaches have been developed based on these classical methods as well. For example, the Mimi-Surv Model built on Cox regression was designed to identify miRNA-mRNA integration set associated with survival time (Kim et al.). Ye et al. proposed a new meta-analysis method to integrate multiple transcriptomic studies and categorize biomarkers by concordant patterns with application to Pan-Cancer studies. Jeong et al. presented a kernel canonical correlation analysis (CCA) method to construct condition specific transcriptional networks. CCA with a positive definite kernel is a well-used method for multiple source data analysis. They employed kernel CCA to embed transcription factors (TFs) and target genes (TGs) into a new space where the correlation of TFs and TGs are reflected. Their approach successfully detected novel TF-TG relations in addition to replicated existing regulatory interactions.

Current methods appropriate for high-dimensional data includes penalized regression models (e.g., LASSO and Ridge regression), kernel-based methods, tree-based methods (e.g., random forest), and latest versatile deep learning models [e.g., Generative Adversarial Networks (GANs)]. In our collection, Ge et al. proposed a modified conditional GANs with new network structures for estimation of individualized treatment effect, which can handle binary and continuous type of treatments. In their framework, LASSO was also used to select biomarkers for optimal treatment selection. Liu and Li proposed a new method for estimation and prediction of heterogeneous restricted mean survival time based on random forest. The application in ovarian cancer showed improved prediction performance vs. existing methods.

With many powerful tools in the field, it is always interesting to evaluate their strengths and appropriate usage. Källberg et al. compared 13 feature selection methods for their ability to identify a subset of genes that can be used to accurately classify cancer subtypes based on gene expression data. Each of the feature selection techniques was applied to four human cancer data sets with known subtypes, enabling accuracy assessment. Their findings demonstrated that the feature selection methods based on modality outperformed the most commonly used approach of selecting the genes with the highest variability.

In addition to the applications in cancer, Zhou et al. used gene expression data and 6 machine learning methods to predict the 3-year survival risk for patients having heart failure with preserved ejection fraction (HFpEF). In their result, the kernel partial least squares with the genetic algorithm (GA-KPLS) outperformed penalized regression, random forest, support vector machine (SVM), and logistic regression. Jiang et al. employed mendelian randomization (MR) and meta-analysis to study the causal relationship between alcohol consumption and risk of autoimmune inflammatory diseases from totaling 1 million individuals' genetic data. With the enormous genetic data and comprehensive analysis, they noted an overall null association between alcohol consumption and common autoimmune inflammatory disorders.

As summarized above, this collection of original research papers presents a significant amount of progress made in the integrative analysis of clinical and (multi-)omics cancer data, prediction in cancer diagnosis/survival/progression, statistical, and machine learning methods for high-dimensional data analysis. While the generation of data has far outpaced our ability to make sense of those data, further development and application of statistical and machine-learning methods are required for the analysis of contemporary genetics in cancer and other diseases.

## Author Contributions

All authors listed have made a substantial, direct and intellectual contribution to the work, and approved it for publication.

## Conflict of Interest

The authors declare that the research was conducted in the absence of any commercial or financial relationships that could be construed as a potential conflict of interest.

## Publisher's Note

All claims expressed in this article are solely those of the authors and do not necessarily represent those of their affiliated organizations, or those of the publisher, the editors and the reviewers. Any product that may be evaluated in this article, or claim that may be made by its manufacturer, is not guaranteed or endorsed by the publisher.

